# Characterization of Nutritional, Physicochemical, and Phytochemical Composition and Antioxidant Capacity of Three Strawberry “*Fragaria* × *ananassa* Duch.” Cultivars (“Primoris”, “Endurance”, and “Portola”) from Western Region of Portugal

**DOI:** 10.3390/foods8120682

**Published:** 2019-12-14

**Authors:** Rui Ganhão, Joaquina Pinheiro, Clara Tino, Hugo Faria, Maria M. Gil

**Affiliations:** 1 MARE-Marine and Environmental Sciences Centre, ESTM, Instituto Politécnico de Leiria, 2520-641 Peniche, Portugal; rganhao@ipleiria.pt (R.G.); maria.m.gil@ipleiria.pt (M.M.G.); 2 Escola Superior de Turismo e Tecnologia do Mar, Instituto Politécnico de Leiria, 2520-641 Peniche, Portugal; clara.tino@gmail.com (C.T.); hugo_faria7@sapo.pt (H.F.)

**Keywords:** strawberry, bioactive, quality, cultivar type, phenolics content

## Abstract

In this study, nutritional composition (protein, lipids, carbohydrates, ash, and moisture), physicochemical properties (soluble solid content, titratable acidity, texture and instrumental colour on surface, and internal section), phytochemicals (total phenolic content and anthocyanin content), and antioxidant capacity (DPPH—2,2′-diphenyl-1-picrylhydrazyl radical scavenging capacity and ferric-reducing antioxidant power) of three strawberry (*Fragaria* × *ananassa* Duch.) cultivars (cv. “Primoris”, cv. “Endurance”, and cv. “Portola”) produced in the western region of Portugal (Caldas da Rainha) were evaluated. From the obtained, results no significant differences (*P* > 0.05) in nutritional composition were detected in all of the cultivars; with the exception of lower protein content observed in cv. “Portola” (0.57 g/100 g ± 0.04; *P* < 0.05). Regarding the a* value of whole strawberry fruits, no significant differences (*P* > 0.05) were found in any of the cultivars, which revealed a similar redness. The cv. “Endurance” revealed the highest bioactivity content compared to the other cultivars. Overall, these results provide important information about the high quality of strawberry produced in the western region of Portugal and may be used as a tool for adding value to a functional food in the Mediterranean diet due to the phytochemical composition and nutritional value of strawberry fruits

## 1. Introduction

Strawberry fruits (*Fragaria* × *ananassa* Duch.) belong to the family of Rosaceae and genus *Fragaria*, and the growing area has been increasing all over the world. In Portugal, strawberry production increased from about 2600 tonnes in 2007 to 9347 tonnes in 2017 [1], with the western region being one of the most representative areas for strawberry growing, along with *Ribatejo* and *Algarve* [2]. The “Primoris”, “Endurance”, and “Portola” strawberry cultivars are the most important ones produced in Portugal [2].

Strawberry is a fruit belonging to the Mediterranean diet, appreciated for its sensorial quality and described as having a particular aroma and sweetness in flavour combined with an agreeable texture [3,4]. Another interesting fact concerning this berry is that it has been reported to be an effective enhancer of the oxidative stability of foods, with berries being among the best sources of phenolic compounds [5,6,7]. Besides this phenolic richness, a combination of vitamin C, folate, and other compounds can be found in this fruit [8].

Moreover, the health-promoting effects associated with the consumption of strawberry fruit has been attributed to the abundance of antioxidative phenolic compounds, including anthocyanins, flavonols, and condensed tannins [9]. Therefore, the increase in this fruit production has been associated not only with high consumer acceptance due to its sensory attributes but also with the presence of bioactive compounds recognized to be beneficial for consumer health, such as antioxidative and anti-inflammatory compounds [10,11].

However, during the growth period, strawberry fruit is subject to several factors that compromise the overall quality and bioactive properties of fruits, such as agricultural practices, production region, and climatic conditions, as well as cultivars [12]. Regarding the influence of high temperature on fruit growth, as an example, temperatures close to 30 °C intensify and promote antioxidant activity, mainly the phenolic and anthocyanin compounds [13]. Nevertheless, the phenolic content and composition as well as the antioxidant capacity of strawberries produced in the western region of Portugal are mostly unknown.

In the present study, three strawberry cultivars (cv. “Primoris”, cv. “Endurance”, and cv. “Portola”) produced in the western region of Portugal were assessed in terms of nutritional composition and overall quality through analysis of physicochemical attributes such as colour (L*, a*, b*, and hue), texture, soluble solid content (SSC), and titratable acidity (TA); through analysis of phytochemical properties by phenolic (TPC) and anthocyanin content (AC); and through analysis of antioxidant capacity by DPPH scavenging activity and ferric-reducing power activity (FRAP). Therefore, this research encompasses relevant findings regarding the strawberry cultivar quality produced in Portugal.

## 2. Materials and Methods

### 2.1. Materials

Three strawberry (*Fragaria* × *ananassa* Duch.) cultivars (cv. “Primoris”, cv. “Endurance”, and cv. “Portola”) were obtained from the fruit and vegetable farm Frutas Classe in the western region of Portugal (Caldas da Rainha). The strawberry harvest time/moment was carried out by producers based on commercial maturation (>75% of visual reddish colour) and evaluated through sensory analysis (appearance and absence of mechanical injuries). Three hundred and thirty fruits (≈7 kg) were picked and transported to a laboratory (5 °C) where, after discarding damaged fruits, about 300 fresh and whole strawberries (≈6.6 kg) were classified, as “Extra category” according to the official Portuguese method [14]. Following the selection by cultivar type, they were divided into three groups per three replicates each (*n* = 3 × 30 × 3).

All chemicals and reagents used in the present study were ACS grade (Committee on Analytical Reagent grade). Methanol HPLC grade was purchased from Pronalab (Lisbon, Portugal) and Fisher-Scientific (Loughborough, England); cupric sulfate (II), titanium dioxide, sulfuric acid 95–97%, Folin-Ciocalteu’s phenol reagent, chloride hexahydrate, petroleum ether, and gallic acid (anhydrous) were from Merck (Darmstad, Germany); methyl red, sodium hydroxide pellets, L-tryptophan, sodium sulfate, ammonium sulfate, sodium carbonate, potassium chloride, cyanidin 3-glucoside, silver nitrate, and hydrochloric acid 37% were from Sigma-Aldrich (St. Louis, MO, USA); acetic acid was from ACROS Organics (Gell, Belgium); sodium acetate was from VWR Prolabo Chemicals (Leuven, Belgium); and 2,2-diphenyl-1-picrylhydrazyl (DPPH) was obtained from Alfa Aesa (Ward Hill, MA, USA)

### 2.2. Methods

#### 2.2.1. Analysis of Nutritional Composition

The proximate composition (protein, lipid, carbohydrate, moisture, and ash) of strawberry cultivars was determined by Association of Official Analytical Chemists (AOAC) International methods [15]. The crude protein was ascertained using the Kjeldhal method (*N* × 6.25) according to AOAC Official Method 920.152. The fat was determined by extracting 5 g of sample with petroleum ether using a Soxhlet apparatus according to AOAC Official Method 963.15. The ash content was obtained by incineration at (600 ± 15) °C, and carbohydrates were calculated by difference: 100 − (g moisture + g protein + g fat + g ash). Moreover, total energy was calculated using the following equation:

(1) Energy (kcal)=4×(g protein + g carbohydrate)+9×(g fat)

The proximate values were calculated from three measurements of each samples group and presented as percentages (%).

#### 2.2.2. Analysis of Physicochemical Properties

Titratable acidity (TA) was measured according to the International Standard Organization (1998) [16], based on a potentiometric titration with a standard volumetric solution of sodium hydroxide. Briefly, twenty-five grams of strawberry pulp was diluted in a 250-mL volumetric flask with distilled water. After being mixed, the diluted sample was titrated with a solution of sodium hydroxide (NaOH) at 0.1 M until reaching pH 8.1. Titratable acidity was expressed as grams of citric acid per 100 g of strawberry fruits. Three measurements per sample group were taken.

Strawberry soluble solid content (SSC) was measured after homogenization in a Yellow line DI 25 basic polytron (KA-Werke GmbH & Co.KG, Staufen, Germany) and was measured in a refractometer (DR-A1, ATAGO Co Ltd., Tokyo, Japan). Three determinations per sample group replicate were carried out, and the result were reported as °Brix.

**Figure 1:**
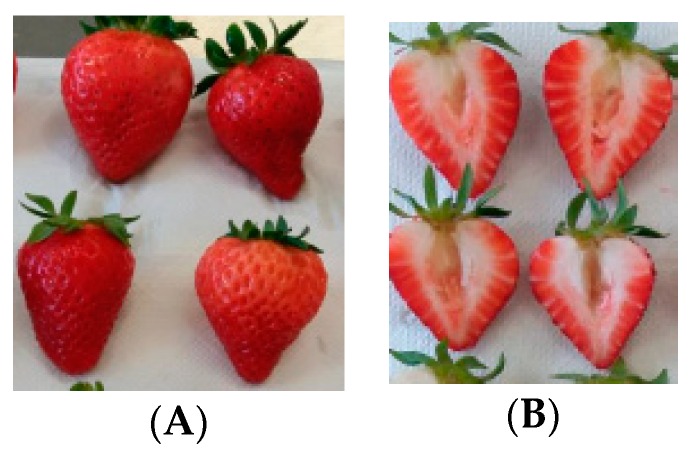
Representation of strawberry fruit area used for colour and texture determination: surface (whole fruits) (**A**) and internal (divided fruits) (**B**).

Colour measurements of superficial and internal strawberry fruits (Figure 1) were evaluated using a tristimulus colorimeter (Minolta chroma Meter, CR-300, Osaka, Japan) with an 8-mm diameter measuring area and a data processor (Minolta chroma Meter, DP-301, Osaka, Japan). The instrument was calibrated using a white standard tile (L* = 97.10, a* = 0.19, and b* = 1.95) and the illuminate D_65_ (2° observer). CIE colour space coordinates, L*a*b* values, were determined, where the L* values represent the luminosity of samples (0 for black to 100 for white) and where a* and b* values indicate the variation of greenness to redness (−60 to +60) and blueness to yellowness (−60 to +60), respectively. Tonality was expressed as hue (°*h*) (Equation (2)) and was calculated as follows:

(2) $$hue=tan^{-1}\;\left(\frac{b^{*}}{a^{*}}\right)$$

Bearing in mind the measurement section of strawberry fruits (Figure 1), ten measurements from each sample group were taken.

The texture was determined according to the modified method described by Tylewicz, Tappi, Genovese, Mozzon, and Rocculi [17]. Strawberry hardness was recorded by the penetration test with a Texture Analyzer (TA.HDi, Stable Microsystem Ltd., Godalming, UK), using a load cell of 5 kg and a stainless steel cylinder probe with a 2-mm diameter. The penetration test was performed at a speed of 1.5 mm/s and a penetration distance of 4 mm. Force–distance curves were recorded and firmness (hardness (N)) and adhesiveness (N/s) were used as indicators of fruit texture. Ten measurements were taken from each sample group in both measurement areas of the fruits, as represented in Figure 1.

#### 2.2.3. Analysis of Phytochemical Quality

Total phenolic content (TPC) was determined using the Folin–Ciocalteu method [18] and adapted for a 96-well plate assay: 20 μL of sample/standard was mixed with 100 μL of diluted Folin–Ciocalteau reagent (1/10, *v*/*v*)), and after 4 min, 80 µL of Na_2_CO_3_ solution (7.5%, (*m*/*v*)) was added. The reaction occurred for 2 h at room temperature, and the absorbance of the mixture was measured at 750 nm using a microplate reader (Synergy H1 Multi-Mode Microplate Reader, BioTek^®^ Instruments, Winooski, VT, USA). Gallic acid was used to prepare a standard calibration curve. TPC results expressed as milligrams of gallic acid equivalents per 100 g of strawberry fruits (mg GAE/100 g) resulted from the average of three measurements per sample group.

Anthocyanin content (AC) of strawberry cultivars was determined by pH difference [19]. Briefly, 3 mL of strawberry samples was mixed with 5 mL of two buffers, pH 1.0 (0.2 M potassium chloride adjusted with chloric acid) and pH 4.6 (0.2 M acetate of sodium adjusted with acetic acid). Both strawberry samples at pH 1.0 and 4.6 were incubated at room temperature for 30 min. The absorbance difference at 530 nm (Synergy H1 Multi-Mode Microplate Reader, BioTek^®^ Instruments, Winooski, VT, USA) of both buffers is proportional to the anthocyanin content. Also, measurements of absorbance at 700 nm were taken into account for corrections due to presence of interfering compounds. The results were expressed as mg cyanidin-3-glucoside per 100 g of strawberry fruits as in the following Equations (3) and (4):

(3) $$Anthocyanin\;concentration\;=\frac{\Delta A\cdot MW\;average\;FD\cdot 1000}{\varepsilon \cdot L}$$

(4) $$\Delta A=\left(A_{530}-\;A_{700}\right)\;pH\;:1.0-\;\left(A_{530}-\;A_{700}\right)pH:4.5\;$$

where ΔA is the difference between absorbance change at 530 and 700 nm in buffers at pH 1 and 4.6; MW_average_ is molecular mass for cyanidin-3-glucoside (449.2 g/mol); FD is the dilution factor; ε is the molar extinction coefficient for cyanidin-3-glucoside (26,900 cm/M); L is the optical path (cm); 1000 is the conversion factor of grams to milligrams; and three measurements were taken from each sample group.

#### 2.2.4. Analysis of Antioxidant Capacity

DPPH scavenging activity was determined according to methodology reported by Custódio et al. [20]. Briefly, 50 µL of sample/standard was reacted with 150 µL of DPPH solution (150 µmol/L) for 1 h in dark at room temperature. Subsequently, the absorbance was measured at 517 nm in a microplate reader (Synergy H1 Multi-Mode Microplate Reader, BioTek^®^ Instruments, Winooski, VT, USA). The percentage of inhibition was expressed as radical scavenging activity (% RSA).

Ferric-reducing antioxidant power (FRAP) was measured by the method of Benzie and Strain [21] and of Bolanos de la Torre, Henderson, Nigam, and Owusu-Apenten [22] adapted for a 96-well plate assay with minor modifications. FRAP reagent was prepared with 0.3 M acetate buffer (pH = 3.6), 10 mM of 2,4,6-tripyridyl-s-triazine (TPTZ) in 40 mM HCl, and 20 mM ferric solution using FeCl_3_ in a 96-well microplate. By freshly mixing acetate buffer, TPTZ, and ferric solutions at ratio of 10:1:1, the final working FRAP reagent was incubated at 37 °C in dark. Briefly, 2 µL of sample was added to 198 µL of FRAP reagent and allowed to stand at 37 °C in dark for 30 min, at which time the increase in absorbance at 593 nm was measured in a microplate reader. Standard curve was performed using FeSO_4_ solution (0.1–1.0 mM), and results were expressed as mM of FeSO_4_ per 100 g of strawberry.

In DPPH scavenging activity and ferric-reducing antioxidant power (FRAP) methodologies, three measurements from each sample group were taken.

### 2.3. Statistical Analysis

In this study, the obtained data are expressed as mean values and standard deviation (SD). All data underwent analysis of variance (ANOVA) using Statistica™ v.8.0 Software from Statsoft (Tulsa, OK, USA) [23]. Statistically significant differences (*P* < 0.05) between sample groups of strawberry cultivars were determined according to Tukey HSD (Honestly Significant Difference). Pearson correlation coefficients were also generated between the quality attributes evaluated: nutritional, physicochemical, phytochemical, and antioxidant capacity.

## 3. Results and Discussion

In accordance with Table 1, the studied strawberry cultivars have been defined as “Extra category” fruits based on uniformity, brightness, and red colour typical of fruits. This evaluation was performed taking into account the Portuguese Regulation, Ministerial Order No. 90 (1988) [14], concerning strawberry quality for consumption. Also, the equatorial section of all strawberry fruits fits the minimum of 25 mm, validating the high quality of the samples.

### 3.1. Evaluation of Nutritional Composition

Table 2 presents the nutritional composition (moisture, protein, fat, carbohydrate, and ash) of the studied strawberry cultivars (cv. “Primoris”, cv. “Endurance”, and cv. “Portola”).

The strawberry cultivars showed no significant difference (*P* > 0.05) in nutritional composition with the exception of protein content, where the cv. “Portola” denotes a lower value (0.570 ± 0.033%), *P* < 0.05) compared to cv. “Primoris” and cv. “Endurance”. The carbohydrates ranged between 9.110% and 12.430% with no significant difference (*P* > 0.05) between the three strawberry cultivars.

These values are higher than 5.30 g and 7.68 g of carbohydrates per 100 g of fresh strawberry announced by the platform of food information in Portugal [24] and considered by the Nutrient Database of US Department of 2018 [25], respectively.

In general, the nutritional composition of the three cultivars can be considered to be within the expected range for this fruit, according to previous studies [26,27]. However, environmental factors [28,29] greatly influence the chemical characteristics of fruits and, consequently, their antioxidants [30]. Overall, the nutritional composition of strawberries produced in Portugal play an important role in the food choices of those looking to include safe, healthy, and nutritious foods in their diet.

### 3.2. Evaluation of Physicochemical Properties

Physicochemical quality of strawberry cultivars produced in Portugal are shown in Table 3.

#### 3.2.1. Titratable Acidity

The titratable acidity of strawberry fruits results from organic and nonvolatile acids, which influence the cellular pH [31]. By observing titratable acidity among strawberry samples, a significant difference (*P* < 0.05) was observed in all the fruit’s cultivars. The cv. “Portola” and cv. “Primoris” attained the lowest and highest values of titratable acidity, (0.572 ± 0.047) g citric acid/100 g and (0.809 ± 0.011) g citric acid/100 g, respectively. The study reported by Lan, Zhang, Ahmed, Qin, and Liu [3] reveals that strawberry fruits produced in a local farm in China had a lower value of titratable acidity (0.68 g citric acid/100 g) than cv. “Primoris” and cv. “Endurance” cultivars. According to the literature, a decrease in titratable acidity was expected in postharvest fruit due to use of organic acids as substrates in respiration [3,32]. Additionally, when there was a reduction in organic acids, the fruits’ senescence was initiated [32]. This evolution in organic acids was responsible for changes in total acidity, which is very high in the phase of fast fruit growth.

Furthermore, differences between titratable acidity of strawberry cultivars could be possible due to differences in maturity at harvest, to differences between the growing conditions, to environmental factors, and to processing techniques of various fruits [32,33,34].

#### 3.2.2. Soluble Solid Content

The main compounds of soluble solid content of fruits are organic acids and sugar, with the sweetness depending on sugar content [35]. A strawberry’s initial metabolic process converts carbohydrates to sugars and other soluble compounds, which increases total soluble solids. As such, a decrease in soluble solid content was obtained through the next step: sucrose hydrolysis that reduced sugars [3]. Nonetheless, at the same maturation stage of strawberry cultivar, cv. “Endurance” showed an increase of ca. 40% compared to cv. “Primoris” (6.82 ± 0.37) °Brix, as shown in Table 3. Considering the study reported by Cao, Guan, Dai, Li, and Zhang [36], a significant difference (*P* < 0.05) between soluble solid content of two strawberry cultivars was attained, about 2 °Brix and 4.3 °Brix, in a German cultivar (cv. Brandenburg) and an American cultivar (cv. Sweet Charlie), respectively. Indeed, the strawberry SSC is influenced by several factors, including genetics, climate, water management, and other cultivation practices as referenced by Wold and Opstad [37].

The correlation between titratable acidity and soluble solid content in different strawberry cultivar was analysed, and the results showed that both quality parameters were positive correlated with coefficient *R*^2^ = 0.6910 (*P* = 0.0001).

#### 3.2.3. Colour

During the strawberry postharvest, the main quality attributes that contribute towards consumer acceptability are the attractive red colour and the sweetness and fruity flavour.

Differences between the fruit zone colour measurements of three strawberry cultivars (cv. “Endurance”, cv. “Primoris”, and cv. “Portola”) can be observed in Table 3 and Figure 1.

Regarding the fruits’ surface zone colour, no significant difference (*P* > 0.05) was denoted in any of the strawberry cultivars, which revealed the typical red colour of these fruits. By contrast, the interior of cv. “Portola” recorded the lowest a* value (18.6 ± 0.6, *P* < 0.05), describing less redness in this particular cultivar of the fruit.

The CIE L*a*b* coordinates observed in cv. “Endurance” were consistent with colour reported by Garrido-Bigotes et al. [38] for cv. “Aromas” produced in Chile. This similarity in colour between strawberry cultivars produced in different countries could be associated with the same ripening stage. Despite the changes in strawberry colour throughout maturity and postharvest period (darkness and less vibrant), these quality attributes were not considered a limiting factor for strawberry shelf-life [32].

#### 3.2.4. Texture

Fruit firmness is one important quality attribute that needs to be measured and controlled, since during postharvest, a softening occurs, which influences the consumer acceptance [39]. Strawberry firmness obtained on surface and internal fruit zones can be observed in Table 3.

Comparing the studied strawberry, produced in the western region of Portugal, cv. “Endurance” exhibited the lowest value of firmness in both fruit measurement sections, (0.612 ± 0.270) N and (0.431 ± 0.240) N in surface and internal fruit zones, respectively. This significant difference (*P* < 0.05) was expressed in about 50% of firmness compared to the values obtained in cv. “Primoris” and cv. “Portola”. These findings highlight the softening texture of this strawberry cultivar. Internal strawberry firmness denoted a reduction in maximum force, 20% lower in cv. “Primoris” and “Portola” than observed in the surface section in both fruits. The strawberry cv. “Portola” has a similar value of firmness to that observed by Ramos et al. [39] in the same fruit cultivar produced in Chile. In this study, a comparative evaluation was performed on cv. strawberry “Portola”, “Camarosa”, “Cristal”, and “Monterey” at different development stages (green, white, 50%, and ripe), where fruit firmness below 1.5 N was achieved at the ripe maturity stage. Previous works have been reporting the effects of essential nutrients during fruit growth and relation to overall quality [40]. During the fruits’ growth, the addition of essential nutrients is important since calcium plays an important role in cell integrity (maintenance of cell permeability and cell division) that directly influences the quality such as strawberry firmness [41], which may be a possible effect on the difference in texture of the strawberries studied. The correlation between moisture and fruit firmness previously demonstrated by Lan et al. [3] was not evident in our study. The “Portola” cultivar showed higher moisture content, and a maximum force as also observed in cv. “Primoris”. However, these findings were not statistically different (*P* > 0.05) in both evaluated texture attributes regarding the moisture and firmness achieved in three fruit cultivars (cv. “Portola”, “Primoris”, and “Endurance”).

### 3.3. Evaluation of Phytochemical Quality

Strawberry belongs to a phytochemical-rich fruits group that is associated with a reduction in the risk of several diseases due essentially to its phenolic content. One of the most important polyphenol compounds investigated in strawberry fruits is anthocyanin [26] due to its health benefits and correlation with red colour, which are the main attributes for assessing strawberry quality [31,42].

The phytochemical content expressed as total phenolic (TPC) and anthocyanin content (AC) of three strawberry cultivars is shown in Table 4.

#### 3.3.1. Phenolic Content

All the studied strawberry cultivars presented a significant (*P* < 0.05) phenolic content, which ranged from 607 to 1314 mg GAE/100 g. In the literature, higher phenolic content is considered to be favourable, since it is an indicator of natural health-promoting bioactive compounds [43].

The strawberry cv. “Portola” exhibited the lowest value of TPC (605.698 ± 28.118) mg GAE/100 g, *P* < 0.05) when compared to cv. “Primoris” and cv. “Endurance” (942 ± 96) and (1314 ± 89) mg GAE/100 g, respectively). Furthermore, our results are similar to those reported by Lester, Lewers, Medina, and Saftner [44] in the same strawberry cultivar (“Portola”) grown on a conventional farming operation in Maletto and from a commercial plantation in Tudla in another Mediterranean country: Italy [45]. In this study, strawberries produced in Maletto (1300 mg GAE/100 g) and Tudla (900 mg GAE/100 g) reveal identical phenolic content to that obtained in “Endurance” and “Primoris”. According to Hakkinen and Torronen [46], fruit cultivar affects the phenolic content of strawberry, which is dependent on production location and the time of year. The scientific literature available for strawberries produced in Portugal is noticeably scarce. In fact, for the first time, this study reports considerably high levels of phenolic compounds in cultivars produced in the western region of Portugal in comparison to other countries.

#### 3.3.2. Anthocyanin Content

Total anthocyanins followed the expected pattern as considerable amounts are mainly found in the red fruit [47]. The most common anthocyanins in red or purple coloured fruits are cyanidin glycosides followed by delphinidin glycosides [48]. Anthocyanin concentration expressed as cyanidin-3-glucoside was revealed to be significantly different (*P* < 0.05) in cv. “Endurance” ((2.411 ± 0.009) mg/100 g) when compared to cv. “Primoris” and cv. “Portola” ((2.09 ± 0.01) and (2.18 ± 0.02) mg/100 g respectively). In this study, the cv. “Endurance” demonstrated a correlation between the anthocyanin content and phenolic content. On the other hand, the studied strawberry cultivars presented a higher AC compared to *Fragaria chiloensis* Mill, known as Chilean strawberry, that contained a lower AC of 1.10 mg/100 g and 1.16 mg/100 g in 2011 and 2012, respectively, as reported by Saavedra et al. [49].

In agreement with the results described above for TPC, the anthocyanin content of cv. “Endurance” was clearly higher than that of the other cultivars. In a recent study, Elisia, Hu, Popovich, and Kitts [50] showed that the presence of cyanidin-3-glucoside in *Rubus ulmifolius* is a major contributor to the antioxidant capacity of this fruit. However, the presence of anthocyanins and their antioxidant properties can vary considerably between species and fruit cultivars [51,52].

Strawberry fruits prove to be an excellent and natural source of dietary anthocyanins that can lead to health benefits [53,54].

### 3.4. Evaluation of Antioxidant Capacity

For the evaluation of food antioxidant capacity, different methodologies are used to obtain valid results because antioxidant compounds present different reactions of mechanisms with possible synergistic interactions [55,56] depending on the type of assay employed. Consequently, the antioxidant capacity of the strawberry cultivars was assessed by the DPPH and the FRAP assays (Table 4).

#### 3.4.1. DPPH Radical Scavenging Activity

The DPPH free radical scavenging activity of strawberry cultivars ranges from 42.0 ± 2.3% to 51.8 ± 5.1%. The values reported in the current study were generally higher than those reported by Wang, Wang, Ye, Vanga, and Raghavan [25] for the strawberry cultivar, “Seascape” after postharvest high-intensity ultrasound treatment prior to juice extraction. Olsson et al. [57] referenced that the antioxidant capacity of strawberry fruits increased throughout the postharvest period from the unripe to the fully ripe maturity stage. Also, environmental conditions, mainly higher temperatures, lead to significantly increased content of antioxidant capacity in berry fruits, as stated by Moretti, Matos, Calbo, and Sargent [58].

#### 3.4.2. FRAP

As shown in Table 4, a significant difference (*P* < 0.05) in ferric-reducing antioxidant power (FRAP) was observed between strawberry cultivars. The cv. “Endurance” showed the highest value ((72.4 ± 8.1) Eq mM FeSO_4_/100 g) when compared to cv. “Primoris” ((58.8 ± 0.9) Eq mM FeSO_4_/100 g) and “Portola” ((38.2 ± 1.4) Eq mM FeSO_4_/100 g). The study developed by Rekika, Khanizadeh, Deschênes, Levasseur, and Charles [59] found a correlation between the anthocyanin content and antioxidant capacity of some selected strawberry genotypes. In the current study, a positive correlation (*R*^2^ = 0.9831, *P* = 0.017) was found between the FRAP and anthocyanin content for the cv. “Endurance” only but not for the other two cultivars. Overall, cv. “Endurance” strawberry recorded the highest TPC and anthocyanin contents consistent with the highest antioxidant activities, especially for FRAP (Table 4). In general, fruits with the highest TPC showed the highest antioxidant activity against the DPPH radical. However, the correspondence between the TPC and the antioxidant activity against DPPH is not so obvious for these cultivars.

On the other hand, the Folin–Ciocalteu assay gives a crude estimate of the total phenolic compounds present in an extract. Moreover, different phenolic compounds respond differently to this assay depending on the number of phenolic groups they contain. Thus, the TPC does not necessarily incorporate all the antioxidants that may be present in an extract. Hence, this may explain the lack of correlation between the TPC and some methods of assessing antioxidant activity for certain fruits.

These results are also in good agreement with the findings of Wang et al. [25]; of Ganhão et al. [33], and of Nowicka et al. [42], who reported such positive correlations between TPC and antioxidant capacity in different fruits and vegetables.

## 4. Conclusions

In this study, a characterization of the most important strawberry cultivars (“Primoris”, “Endurance”, and “Portola”) produced in the western region of Portugal was performed. All the studied strawberry cultivars exhibited a natural source of health-promoting properties through phytochemical and antioxidant screening and testing for nutritional value. The “Endurance” strawberry cultivar was characterized by the highest level of antioxidant compounds, expressed by total phenolic and anthocyanin content. These antioxidant compounds may have useful applications as functional ingredients in foods. In this sense and for the development of functional ingredients, the economic value of this cultivar produced in Portugal should be enhanced, with interest for producers and consumers. Identification should be made of phenolic compounds as biomarkers to better understand the influence of the bioavailability of these compounds on promoting good health. Moreover, these findings will be important for promoting the Mediterranean diet and for consumers who choose to have a daily intake of foods rich in antioxidants.

## Figures and Tables

**Table 1:** Classification of strawberry cultivars (cv. “Primoris”, cv. “Endurance”, and cv. “Portola”) produced in the western region of Portugal (Caldas da Rainha).

Cultivar	Calibre (mm)	Height (mm)
“Primoris”	48.9 ± 5.3	34.9 ± 2.9
“Endurance”	41.7 ± 4.8	36.9 ± 3.7
“Portola”	42.2 ± 5.4	46.4 ± 4.8

Classification was performed according to Ministerial No 90/1988—Annex III—specification of strawberry quality. This document contains the specifications of strawberry fruits regarding the genus *Fragaria L*. for fresh consumption. Data are presented as “mean ± standard deviation”.

**Table 2:** Nutritional composition of strawberry cultivars (cv. “Primoris”, cv. “Endurance”, and cv. “Portola”) produced in the western region of Portugal.

Component (%)	cv. “Primoris”	cv. “Endurance”	cv. “Portola”
Moisture	86.3 ± 2.0 ^a^	88.2 ± 1.1 ^a^	89.8 ± 2.1 ^a^
Crude Protein	0.730 ± 0.057 ^a^	0.720 ± 0.041 ^a^	0.570 ± 0.033 ^b^
Crude Fat	0.130 ± 0.008 ^a^	0.150 ± 0.024 ^a^	0.140 ± 0.024 ^a^
Carbohydrate	12.4 ± 1.7 ^a^	10.5 ± 1.0 ^a^	9.11 ± 1.79 ^a^
Ash	0.430 ± 0.016 ^a^	0.460 ± 0.024 ^a^	0.430 ± 0.024 ^a^
Energy (kcal)	53.8	46.0	40.0

Data are expressed as “mean ± standard deviation” of triplicate measurements. ^a,b^ In the same line, different letters indicate significant difference (*P* < 0.05, Tukey test) between strawberry cultivars.

**Table 3:** Characterization of physicochemical quality (titratable acidity (TA), soluble solid content (SSC), colour, and texture) of the studied strawberry cultivars.

Physicochemical Properties	cv. “Primoris”	cv. “Endurance”	cv. “Portola”
**Titratable acidity** (g citric acid/100 g)	0.809 ± 0.011 ^a^	0.739 ± 0.009 ^a^	0.572 ± 0.047 ^c^
**Soluble solid content** (°Brix)	6.82 ± 0.37 ^b^	8.51 ± 0.34 ^a^	4.89 ± 0.25 ^c^
**Colour**			
Superficial zone (surface)			
L*	42.3 ± 3.6 ^a^	36.0 ± 3.5 ^c^	38.5 ± 2.7 ^b^
a*	33.3 ± 1.8 ^a^	34.5 ± 2.1 ^a^	32.8 ± 1.7 ^a^
b*	28.8 ± 5.1 ^a^	24.1 ± 3.2 ^b^	30.4 ± 3.7 ^a^
*°h*	40.5 ± 5.2 ^a^	34.8 ± 2.8 ^b^	42.7 ± 3.7 ^a^
Internal zone (interior)			
L*	32.5 ± 0.3 ^b^	37.2 ± 1.8 ^a^	36.4 ± 1.2 ^a^
a*	21.6 ± 0.7 ^a^	21.7 ± 1.1 ^a^	18.6 ± 0.6 ^b^
b*	17.6 ± 0.4 ^b^	17.2 ± 1.1 ^b^	18.9 ± 0.7 ^a^
*°h*	39.2 ± 0.9 ^b^	38.5 ± 1.2 ^c^	45.5 ± 0.9 ^a^
**Texture**			
Superficial zone (surface)			
Hardness (N)	1.23 ± 0.39 ^a^	0.612 ± 0.270 ^b^	1.26 ± 0.43 ^a^
Adhesiveness (N/s)	1.80 ± 0.58 ^a^	0.871 ± 0.321^b^	1.87 ± 0.69 ^a^
Internal zone (interior)			
Hardness (N)	0.950 ± 0.299 ^a^	0.431 ± 0.240 ^b^	0.998 ± 0.306 ^a^
Adhesiveness (N/s)	1.69 ± 0.48 ^a^	0.748 ± 0.419 ^b^	1.73 ± 0.50 ^a^

Data are expressed as “mean ± standard deviation”. ^a,b^ In the same line, different letters indicate significant differences (*P* < 0.05, Tukey test) between strawberry cultivars.

**Table 4:** Characterization of phytochemical quality (Total phenolic content—TPC and anthocyanin—AC) and antioxidant capacity (DPPH—2,2′-diphenyl-1-picrylhydrazyl and ferric-reducing power activity—FRAP) of the strawberry cultivars studied.

	cv. “Primoris”	cv. “Endurance”	cv. “Portola”
**Phytochemical**			
Total phenolic content (mg gallic acid equivalent GAE/100 g)	942 ± 96 ^b^	1314 ± 89 ^a^	607 ± 28 ^c^
Anthocyanin content (mg/100 g)	2.09 ± 0.01 ^b^	2.41 ± 0.01 ^a^	2.18 ± 0.02 ^b^
**Antioxidant Capacity**			
DPPH radical scavenging activity (RSA, %)	51.8 ± 5.1 ^a^	51.1 ± 5.2 ^a^	42.0 ± 2.3 ^a^
FRAP (Eq mM FeSO_4_/100 g)	58.8 ± 0.9 ^b^	72.4 ± 8.1 ^a^	38.2 ± 1.4 ^c^

Data are expressed as “mean ± standard deviation”. ^a,b,c^ In the same line, different letters indicate significant differences (Tukey test, *P* < 0.05) between strawberry cultivars.
